# Surgery for extraforaminal lumbar disc herniation: a single center comparative observational study

**DOI:** 10.1007/s00701-020-04313-w

**Published:** 2020-04-13

**Authors:** Samuel B. Polak, Mattis A. Madsbu, Vetle Vangen-Lønne, Øyvind Salvesen, Øystein Nygaard, Tore K. Solberg, Carmen L. A. M. Vleggeert-Lankamp, Sasha Gulati

**Affiliations:** 1 Department of Neurosurgery, Leiden University Medical Center, Leiden, Netherlands; 2 Department of Neurosurgery, St. Olavs University Hospital, Trondheim, Norway; 3 Department of Neuroscience, Norwegian University of Science and Technology (NTNU), Trondheim, Norway; 4 Department of Public Health and General Practice, Norwegian University of Science and Technology (NTNU), Trondheim, Norway; 5 The Norwegian National Registry for Spine Surgery, University Hospital of Northern Norway (UNN), Tromsø, Norway; 6 Department of Neurosurgery, University Hospital of Northern Norway (UNN), Tromsø, Norway; 7 National Advisory Unit on Spinal Surgery, St. Olavs University Hospital, Trondheim, Norway

**Keywords:** Intervertebral disc displacement, Neurosurgery, Orthopedics, Sciatica

## Abstract

**Background:**

Surgery on extraforaminal lumbar disc herniation (ELDH) is a commonly performed procedure. Operating on this type of herniation is known to come with more difficulties than on the frequently seen paramedian lumbar disc herniation (PLDH). However, no comparative data are available on the effectiveness and safety of this operation. We sought out to compare clinical outcomes at 1 year following surgery for ELDH and PLDH.

**Methods:**

Data were collected through the Norwegian Registry for Spine Surgery (NORspine). The primary outcome measure was change at 1 year in the Oswestry Disability Index (ODI). Secondary outcome measures were quality of life measured with EuroQol 5 dimensions (EQ-5D); and numeric rating scales (NRSs).

**Results:**

Data of a total of 1750 patients were evaluated in this study, including 72 ELDH patients (4.1%). One year after surgery, there were no differences in any of the patient reported outcome measurements (PROMs) between the two groups. PLDH and ELDH patients experienced similar changes in ODI (− 30.92 vs. − 34.00, *P* = 0.325); EQ-5D (0.50 vs. 0.51, *P* = 0.859); NRS back (− 3.69 vs. − 3.83, *P* = 0.745); and NRS leg (− 4.69 vs. − 4.46, *P* = 0.607) after 1 year. The proportion of patients achieving a clinical success (defined as an ODI score of less than 20 points) at 1 year was similar in both groups (61.5% vs. 52.7%, *P* = 0.204).

**Conclusions:**

Patients operated for ELDH reported similar improvement after 1 year compared with patients operated for PLDH.

## Introduction

Sciatica due to lumbar disc herniation (LDH) is the most common indication for spinal surgery [20]. Lumbar microdiscectomy is the most common procedure for LDH and also one of the most frequently performed neurosurgical procedures [1, 3]. Most of LDH are of the paramedian type, but approximately 7–12% of herniations of lumbar discs have been reported to be extraforaminal [2, 18, 22]. The symptoms of extraforaminal lumbar disc herniations (ELDH) are similar to paramedian LDHs (PLDH), namely, radicular pain in the legs [19]. However, leg pain caused by ELDH is believed to give worse pain experience [21]. The current practice for patients suffering from LDH, both paramedian and extraforaminal, is to undergo surgical treatment when pain is intolerable, persists after a period of conservative treatment or when there are disabling neurological deficits [4]. Surgery on ELDH is experienced by surgeons to be more challenging than PLDH operations [1, 9]. However, there are limited data on clinical outcomes following surgery for ELDH. Therefore, research is warranted in order to advice patients and to make evidence-based decisions about the treatment for ELDH.

The aim of this study was to assess clinical outcomes of patients 1 year after surgery for ELDH, compared with those operated for PLDH using data from the Norwegian Registry for Spine Surgery (NORspine).

## Materials and methods

### Study population

Data were collected through NORspine, a comprehensive registry for quality control and research [13]. According to the Norwegian Directorate of Health, approximately 63% of all patients who underwent lumbar spine surgery in Norway during the study period were included in NORspine. Participation in the registration by providers or patients was not mandatory, nor was participation required as a necessary condition for a patient to gain access to health care or for a provider to be eligible for payment. Follow-up time from the date of the operation (baseline) was 1 year. This research was conducted as a single center study, allowing radiological validation of the diagnoses. All patients were operated in the time period between 2013 and 2016 by experienced senior neurosurgeons using the same technique, at one regional university hospital (St. Olavs Hospital, Trondheim, Norway).

Both ELDH and PLDH were operated with a 3–4 cm midline incision and the use of the operating microscope. All patients received preoperative antibiotic prophylaxis and intraoperative fluoroscopy was routinely performed. Surgery for ELDH was performed with a midline incision and ipsilateral paravertebral muscle retraction using Caspar retractors to expose the lateral pars and facet. If necessary, the lateral pars and upper part of the facet joint were resected by using surgical punches or a long, angled drill. The compressed nerve root was then typically retracted superiorly to remove the disc herniation. Surgery for PLDH was performed using a midline incision, ipsilateral paravertebral muscle retraction using Caspar retractors with arcotomy and medial facetectomy if necessary, flavectomy and removal of the disc herniation.

We considered all patients as eligible if they had a definitive diagnosis of symptomatic LDH, planned surgery for either a paramedian or extraforaminal LDH, and inclusion in the NORspine registry. Patients were excluded if they had undergone previous spinal surgery, if they had coexisting spinal deformations such as spondylolisthesis and/or scoliosis, or if they had undergone fusion surgery.

### Primary outcome measure

Changes in disease-specific health-related quality of life were measured using Oswestry Disability Index (ODI) version 2.0 translated into Norwegian and validated for psychometric properties [5, 6, 23]. ODI contains 10 questions on limitations of activities of daily living. Each variable is rated in a 0- to 5-point scale, summarized, and converted into a percentage score. Scores range from 0 to 100, with a lower score indicating less severe pain and disability. Since patients are declared as minimally disabled when the ODI score is less than 20 points [25], we also looked at the amount of patients achieving this score after 12 months.

### Secondary outcome measure

Changes in generic health-related quality of life were measured with the generic EuroQol 5 dimensions (EQ-5D) instrument between baseline and 1-year follow-up. The EQ-5D questionnaire evaluates the generic quality of life along five dimensions, including mobility, self-care, usual activities, pain/discomfort, and anxiety/depression. For each dimension, three levels of problems can be indicated (no, moderate, or severe). Intensity of pain was graded in two separate 0–10 numerical rating scales (NRS) for back pain and leg pain where 0 equals no pain and 10 represents the worst imaginable or ever experienced pain by the patient [8]. The NRS pain scales and ODI have shown good validity and are frequently used in research on back pain [6]. We also compared duration of surgical procedures, length of hospital stays, repeated surgery at the index level within 3 months of surgery, and surgical complication rates.

### Data collection and registration by the NORspine registry protocol

On admission for surgery, the patients completed the baseline questionnaire, which included questions about demographics and lifestyle issues in addition to the patient reported outcome measures (PROMs). During the hospital stay, using a standard registration form, the surgeon recorded data concerning diagnosis, previous lumbar spine surgery, comorbidity, *American Society of Anesthesiologists* (ASA) grade, image findings, and surgical approach and procedure. The surgeons provided data on the following possible complications and adverse events to the NORspine registry: intraoperative hemorrhage requiring blood replacement, postoperative hematoma requiring repeated surgery, unintentional durotomy, nerve injury, cardiovascular complications, respiratory complications, anaphylactic reactions, and wrong level surgery. Patients reported the following complications if they occurred within 3 months of surgery: wound infection, urinary tract infection, pneumonia, pulmonary embolism, and deep venous thrombosis. A questionnaire with pre-stamped return envelopes was distributed to patients by regular mail at 3 months and 1 year after surgery, completed at home by the patients, and returned to the central registry unit. The patients who did not respond received one reminder with a new copy of the questionnaire. The patients completed preoperative and follow-up questionnaires without any assistance from the surgeon or other staff from the treating hospital. In order to identify the exact number of patients operated for extraforaminal disc herniation, we retrospectively reviewed all patient journals and radiological imaging for selected cases.

### Statistical analysis

Statistical analyses were performed with the use of SPSS version 25.0 (IBM Corporation, Chicago, Illinois, USA). Statistical significance level was defined as *P* < 0.05 on the basis of a two-sided hypothesis test with no adjustments made for multiple comparisons. Central tendencies are presented as means when normally distributed and as medians when skewed. We used Chi-square tests for categorical variables. Baseline and 1-year scores were compared with the paired-samples t test. Mean change scores between the groups were analyzed with independent-samples t-test and mixed linear models on all available data. A multiple linear regression model was applied to assess the relationship between the change in ODI score at 1 year (dependent variable) and ELDH, controlling for potential confounders [7, 10–12]. In this regression model, patients were categorized according to their body mass index (BMI) as normal (≤ 30 kg/m^2^, reference), or obesity (> 30 kg/m^2^) (i.e., as “dummy variables”). Due to a strong nonlinear relationship between preoperative ODI and the dependent variable, patients were categorized according to the preoperative ODI score: ODI 0–20 (minimal disability, reference), ODI 21–40 (moderate disability), ODI 41–60 (severe disability), ODI 61–80 (crippled), or ODI 81–100 (bed-bound) (i.e., as “dummy variables”).

### Missing data

Missing data were handled with mixed linear models. This strategy was in line with studies showing that it is not necessary to handle missing data using multiple imputations before performing a mixed model analyses on longitudinal data [10, 26].

## Results

### Study population

The 1750 participants enrolled in this study included 72 patients with ELDH (4.1%) and 1678 patients with PLDH (95.9%). In total, 1184 patients (67.5%) completed the 12 months ODI follow-up, including 55 ELDH patients (76.4%) and 1124 in the PLDH group (67.0%) (*P* = 0.096). Baseline characteristics, surgical treatment, and comorbidities are summarized in Table 1. A significantly higher number of patients with ELDH reported a preoperative duration of sciatica less than 3 months compared with patients operated for PLDH (29.7% [PLDH] vs. 50% [ELDH], *P* = 0.001). The duration of 3–12 months of preoperative sciatica was seen more frequently in the PLDH group (44.2% vs. 28.8%, *P* = 0.016). Duration of sciatica over 12 months was similar in both groups (26% vs. 21.2%, *P* = 0.473).

**Table 1:** Demographic characteristics, coexisting illnesses, and measures of health status for both groups of Patients

Variable	Paramedian LDH	Extraforaminal LDH	*P* value
*n* (%)	1678 (95.9)	72 (4.1)	
Age (years), median (range)	45.0 (16–87)	56.5 (25–85)	< 0.001
Female sex, *n* (%)	710 (42.3)	21 (29.2)	0.027
Married or partner, *n* (%)	1230 (74.4)	54 (75.0)	0.911
Attended college, *n* (%)	610 (36.6)	23 (32.9)	0.525
Mean body mass index	26.91	26.86	0.940
Current smoker, *n* (%)	481 (28.9)	24 (34.3)	0.332
Coexisting spinal stenosis in the operated level	154 (9.2)	6 (8.3)	0.697
Comorbidity, *n* (%)	524 (31.2)	24 (33.3)	0.706
Cardiovascular disease	94 (5.6)	5 (6.9)	0.629
Cerebrovascular disease	17 (1.0)	2 (2.8)	0.157
Vascular claudication	2 (0.1)	0 (0.0)	0.769
Diabetes mellitus	62 (3.7)	1 (1.4)	0.304
Osteoporosis	3 (0.2)	0 (0.0)	0.720
Knee and/or hip osteoarthritis	39 (2.3)	3 (4.2)	0.317
Chronic neurologic disease	18 (1.1)	0 (0.0)	0.377
Chronic musculoskeletal pain	43 (2.6)	2 (2.8)	0.910
Cancer	19 (1.1)	3 (4.2)	0.024
Rheumatoid arthritis	8 (0.5)	0 (0.0)	0.557
Ankylosing spondylitis	8 (0.5)	1 (1.4)	0.289
Other rheumatic diseases	24 (1.4)	1 (1.4)	0.997
Depression and/or anxiety	41 (2.4)	1 (1.4)	0.567
ASA grade > 2	179 (10.7)	7 (9.7)	0.798
Mean preoperative ODI	48.63	53.75	0.030
Mean preoperative EQ-5D	0.22	0.15	0.095
Preoperative diagnostic imaging, *n* (%)			
Preoperative MRI	1627 (97)	67 (93.1)	0.077
Preoperative CT	90 (5.5)	3 (4.2)	1.000
Level of surgery, *n* (%)			
L2-L3	36 (2.1)	5 (6.9)	0.008
L3-L4	144 (8.6)	19 (26.4)	< 0.001
L4-L5	777 (46.3)	25 (34.7)	0.053
L5-S1	708 (42.2)	22 (30.6)	0.050

*ASA*, American Society of Anesthesiologists; *CT*, computed tomography; *EQ-5D*, EuroQol 5 dimensions; *LDH*, lumbar disc herniation; *MRI*, magnetic resonance imaging; *ODI*, Oswestry Disability Index

For the total study population, there was a significant improvement in the ODI score after surgery (− 32.22 points [95% CI, − 30.83 to − 33.61], *P* < 0.001). PLDH and ELDH patients experienced similar changes in ODI (− 30.92 vs. − 34.00, *P* = 0.325).

Patients operated for PLDH showed a lower median age than the ELDH patient population (45.0 years vs. 56.5 years, *P* < 0.001). The control group contained more female participants than the ELDH study population (42.3% vs. 29.2%, *P* = 0.027).

Preoperative ODI scores were significantly lower among the PLDH patients than in the ELDH group (48.63 points vs. 53.75 points, *P* = 0.030). ELDH patients were as likely to achieve less than 20 points on the ODI scale at 1 year compared with PLDH (61.5% vs. 52.74%, *P* = 0.204).

There were no clinically relevant differences between the two groups in outcomes at 1 year regarding all other PROMs (EQ-5D, NRS back pain, and NRS leg pain), presented in Table 2. Similar results were found in the mixed linear model analyses for missing data. Furthermore, there were no differences found in duration of surgery and hospital stay or in complication rates, as outlined in Table 3**.**

**Table 2:** Complete case analysis for ODI

Variable	Paramedian LDH (*n* = 1124)			Extraforaminal LDH (*n* = 55)			Difference in mean change between groups (95% CI)	*P* value
	Baseline	1 year	Mean Change	Baseline	1 year	Mean change		
ODI	49.22	17.21	− 32.01	56.43	20.00	− 36.43	4.42 (− 2.18 to 11.0)	0.189
EQ-5D	0.22	0.72	0.50	0.13	0.64	0.50	0.00 (− 0.1 to 0.1)	0.943
Back pain NRS	6.59	2.96	− 3.62	7.33	3.35	− 3.98	0.36 (− 0.5 to 1.3)	0.426
Leg pain NRS	7.12	2.40	− 4.68	7.53	3.09	− 4.43	− 0.24 (− 1.2 to 0.7)	0.610
Mixed linear model analyses								
ODI	48.39	17.48	− 30.92	53.97	19.97	− 34.00	3.08 (− 3.06 to 9.22)	0.325
EQ-5D	0.22	0.72	0.50	0.14	0.65	0.51	0.01 (− 0.12 to 0.10)	0.859
Back pain NRS	6.66	2.97	− 3.69	7.08	3.26	− 3.83	0.14 (− 0.70 to 0.99)	0.745
Leg pain NRS	7.11	2.41	− 4.69	7.45	3.00	− 4.46	− 0.23 (− 1.13 to 0.66)	0.607

(EQ-5D, *n* = 1059 [ELDH = 49]; NRS back pain, *n* = 1147 [ELDH = 52]; NRS leg pain, *n* = 1145 [ELDH = 53])

**Table 3:** Other postoperative outcomes at 1 year

Variable	Paramedian LDH	Extraforaminal LDH	*P* value
Operation time (minutes), mean	65.78	71.89	0.154
Days in hospital, number, mean	1.37	1.57	0.120
Total complications, number (%)	30 (1.8)	1 (1.4)	0.948
Perioperative complications, number (%)	34 (2.0)	1 (1.4)	0.705
Unintentional durotomy	20 (1.2)	0 (0.0)	0.351
Nerve injury	2 (0.1)	0 (0.0)	0.769
Blood replacement or postoperative hematoma	5 (0.3)	1 (1.4)	0.121
Cardiovascular complications	2 (0.1)	0 (0.0)	0.769
Respiratory complications	1 (0.1)	0 (0.0)	0.836
Anaphylactic reaction	1 (0.1)	0 (0.0)	0.836
Wrong level surgery	1 (0.1)	0 (0.0)	0.836
Patient-reported complications within 3 months, number (%)	89 (7.9)	6 (10.9)	0.424
Wound infection	25 (2.2)	3 (5.5)	0.124
Urinary tract infection	35 (3.1)	1 (1.8)	0.587
Pneumonia	7 (0.6)	1 (1.8)	0.291
Pulmonary embolism	3 (0.3)	0 (0.0)	0.702
Deep vein thrombosis	3 (0.3)	0 (0.0)	0.702
Micturition problems	32 (2.8)	1 (1.8)	0.653
Reoperation within 90 days	106 (6.3)	5 (6.9)	0.803

*LDH*, lumbar disc herniation

### Multiple regression analysis

A multiple regression analysis was performed with change in ODI score at 1 year as the dependent variable. A negative value in the outcome corresponds with less pain-related disability. The effect estimates are presented in Table 4**.**

**Table 4:** Multiple regression analysis with change in ODI at 1 year as the dependent variable

	Parameter estimate	95% CI	*P* value
ELDH	− 0.55	− 5.8–4.7	0.839
Smoker	4.9	2.3–7.6	< 0.001
ODI score 21–40 preoperative	− 10.4	− 15.3 to − 5.5	< 0.001
ODI score 41–60 preoperative	− 29.4	− 34.4 to − 24.4	< 0.001
ODI score 61–80 preoperative	− 48.2	− 53.5 to − 43.0	< 0.001
ODI score ≥ 81 preoperative	− 67.1	− 73.2 to − 61.1	< 0.001
Age > 65	4.1	1.0–7.2	0.009
Depression and/or anxiety	6.6	− 1.3–14.6	0.103
ASA grade > 2	1.9	− 1.8–5.6	0.324
Female sex	2.5	0.3–4.8	0.029
Obesity (BMI > 30 kg/m^2^)	3.5	0.7–6.3	0.014

A negative value in the outcome corresponds to less low back pain related disability. *ASA*, American Society of Anesthesiologists; *BMI*, body mass index; *CI*, confidence interval; *ELDH*, extraforaminal lumbar disc herniation; *ODI*, Oswestry Disability Index

There was no significant correlation between the type of LDH diagnosis and the ODI score after 1 year. Preoperative ODI score was the strongest predictor of outcome, as increasing values correlated with improvement at 1 year. Smoking, age ≥ 65 years, female sex, and obesity were identified as independent predictors for less improvement of ODI at 1 year.

## Discussion

This single center observational registry-based study shows that patients operated for extraforaminal lumbar disc herniation experienced similar improvement after 1 year as those who underwent surgery for the more common paramedian lumbar disc herniation. Furthermore, both groups were as likely to achieve a minimal disability, defined as less than 20 points on the ODI scale. In clinical practice, our study suggests that the threshold for surgery for ELDH should be similar to PLDH.

Other studies have shown that a high preoperative ODI score is associated with greater improvement [10, 12]. In this study, patients with ELDH had a significantly higher, but clinically similar ODI score before surgery compared with the PLDH, and the improvement in ODI was similar. This may emphasize the safety of the surgical technique, despite the complexity of the anatomical challenges when operating on ELDH [1, 9].

Although surgery for ELDH is considered more challenging than surgery for PLDH, no differences in postoperative outcomes and complications were observed. This is possibly explained by the experience of the surgeons operating and the similarity of the entry route for ELDH and PLDH [1, 3, 20].

Patients operated for ELDH reported a shorter duration of symptoms before receiving treatment. This could be the result of the higher preoperative pain, which was experienced by the patients with ELDH. In general, surgeons are more prone to operate on patients that experience much pain. Considering the higher amount of pain experienced by ELDH patients, they will probably receive surgery at an earlier time despite the complexity of their disease.

As our study did not include conservatively treated patients, nothing can be said about the results of surgery compared with conservative care. However, previous studies have shown that early surgical care provides more rapid pain relief and is more cost-effective than prolonged conservative treatment in LDH patients, although no significant differences in outcome after 1-year of follow-up [14–17, 27]. Nonetheless, no evidence is provided on surgical versus conservative care in the specific ELDH patient group, requiring future randomized controlled trials.

### Study strengths and limitations

We used specific inclusion and exclusion criteria based on prospective data collection and a relatively large sample size. These factors combined, all strengthens our results. The main limitation in our study is the high number of patients lost to follow-up. However, a previous study on a similar patient population showed no difference between responders and nonresponders [24]. Also, the percentage of patients lost to follow-up in the ELDH group was substantially lower compared with the PLDH group. Another limitation is our rather low number of patients operated for ELDH. Recent updates in the NORspine registration will make it easier to identify patients with ELDH, allowing a multicenter observational study in the future. Complication rates were partly surgeon reported and underestimation therefore cannot be excluded.

## Conclusion

This single center observational study shows that, at 1 year, patients operated for extraforaminal lumbar disc herniation and paramedian lumbar disc herniation reported equivalent improvement. Furthermore, both groups were as likely to achieve what is considered a minimal disability.

## References

[1] Al-Khawaja DO, Mahasneh T, Li JCJJSS (2016) Surgical treatment of far lateral lumbar disc herniation: a safe and simple approach. 2:21–24 10.21037/jss.2016.01.05 PMC5039843 27683691

[2] An HS, Vaccaro A, Simeone FA, Balderston RA, O'Neill D (1990). Herniated lumbar disc in patients over the age of fifty. J Spinal Disord.

[3] Arts MP, Brand R, van den Akker ME, Koes BW, Bartels RH, Peul WC (2009). Tubular diskectomy vs conventional microdiskectomy for sciatica: a randomized controlled trial. JAMA.

[4] Deyo RA, Mirza SK (2016). Clinical practice. Herniated Lumbar Intervertebral Disk. N Engl J Med.

[5] Fairbank JC, Couper J, Davies JB, O'Brien JP (1980). The Oswestry low back pain disability questionnaire. Physiotherapy.

[6] Grotle M, Brox JI, Vollestad NK (2003). Cross-cultural adaptation of the Norwegian versions of the Roland-Morris Disability Questionnaire and the Oswestry Disability Index. J Rehabil Med.

[7] Gulati S, Madsbu MA, Solberg TK, Sorlie A, Giannadakis C, Skram MK, Nygaard OP, Jakola AS (2017). Lumbar microdiscectomy for sciatica in adolescents: a multicentre observational registry-based study. Acta Neurochir.

[8] Jensen MP, Karoly P (2011). Self-report scales and procedures for assessing pain in adults. Handbook of pain assessment.

[9] Lee S, Kang JH, Srikantha U, Jang IT, Oh SH (2014). Extraforaminal compression of the L-5 nerve root at the lumbosacral junction: clinical analysis, decompression technique, and outcome. J Neurosurg Spine.

[10] Madsbu MA, Solberg TK, Salvesen O, Nygaard OP, Gulati S (2017). Surgery for herniated lumbar disk in individuals 65 years of age or older: a multicenter observational study. JAMA Surg.

[11] Madsbu MA, Oie LR, Salvesen O, Vangen-Lonne V, Nygaard OP, Solberg TK, Gulati S (2018). Lumbar microdiscectomy in obese patients: a multicenter observational study. World Neurosurg.

[12] Madsbu MA, Salvesen O, Werner DAT, Franssen E, Weber C, Nygaard OP, Solberg TK, Gulati S (2018). Surgery for herniated lumbar disc in daily tobacco smokers: a multicenter observational study. World Neurosurg.

[13] Nerland US, Jakola AS, Solheim O, Weber C, Rao V, Lønne G, Solberg TK, Salvesen Ø, Carlsen SM, Nygaard ØP, Gulati S (2015) Minimally invasive decompression versus open laminectomy for central stenosis of the lumbar spine: pragmatic comparative effectiveness study. BMJ : British Medical Journal 350 10.1136/bmj.h1603 PMC4381635 25833966

[14] Ng LC, Sell P (2004). Predictive value of the duration of sciatica for lumbar discectomy. A prospective cohort study. The Journal of Bone and Joint Surgery British Volume.

[15] Nygaard OP, Kloster R, Solberg T (2000). Duration of leg pain as a predictor of outcome after surgery for lumbar disc herniation: a prospective cohort study with 1-year follow up. J Neurosurg.

[16] Peul WC, van Houwelingen HC, van den Hout WB, Brand R, Eekhof JA, Tans JT, Thomeer RT, Koes BW (2007). Surgery versus prolonged conservative treatment for sciatica. N Engl J Med.

[17] Peul WC, van den Heout WB, Brand R, RTWM T, Koes BW, Leiden-The Hague Spine Intervention Prognostic Study G (2008). Prolonged conservative care versus early surgery in patients with sciatica caused by lumbar disc herniation: two year results of a randomised controlled trial. BMJ (Clinical Research Ed).

[18] Phan K, Dunn AE, Rao PJ, Mobbs RJ (2016). Far lateral microdiscectomy: a minimally-invasive surgical technique for the treatment of far lateral lumbar disc herniation. Journal of Spine Surgery (Hong Kong).

[19] Porchet F, Fankhauser H, de Tribolet N (1994). Extreme lateral lumbar disc herniation: clinical presentation in 178 patients. Acta Neurochir.

[20] Postacchini F, Postacchini R (2011). Operative management of lumbar disc herniation : the evolution of knowledge and surgical techniques in the last century. Acta Neurochir Suppl.

[21] Samini F, Bahadorkhan G, Ehsaei MR, Kheradmand H (2008). Intraforaminal and extraforaminal far lateral lumbar disc herniation ( a review of 63 cases) %J. Med J Islam Repub Iran.

[22] Siebner HR, Faulhauer K (1990). Frequency and specific surgical management of far lateral lumbar disc herniations. Acta Neurochir.

[23] Solberg TK, Olsen JA, Ingebrigtsen T, Hofoss D, Nygaard OP (2005). Health-related quality of life assessment by the EuroQol-5D can provide cost-utility data in the field of low-back surgery. European Spine Journal : official Publication of the European Spine Society, the European Spinal Deformity Society, and the European Section of the Cervical Spine Research Society.

[24] Solberg TK, Sorlie A, Sjaavik K, Nygaard OP, Ingebrigtsen T (2011). Would loss to follow-up bias the outcome evaluation of patients operated for degenerative disorders of the lumbar spine?. Acta Orthop.

[25] Solberg T, Johnsen LG, Nygaard ØP, Grotle M (2013). Can we define success criteria for lumbar disc surgery? : estimates for a substantial amount of improvement in core outcome measures. Acta Orthop.

[26] Twisk J, de Boer M, de Vente W, Heymans M (2013). Multiple imputation of missing values was not necessary before performing a longitudinal mixed-model analysis. J Clin Epidemiol.

[27] van den Hout WB, Peul WC, Koes BW, Brand R, Kievit J, RTWM T, Leiden-The Hague Spine Intervention Prognostic Study G (2008). Prolonged conservative care versus early surgery in patients with sciatica from lumbar disc herniation: cost utility analysis alongside a randomised controlled trial. BMJ (Clinical Research Ed).

